# Tobacco treatment clinics in urban public housing: feasibility and outcomes of a hands-on tobacco dependence service in the community

**DOI:** 10.1186/s12889-021-11561-7

**Published:** 2021-08-05

**Authors:** Panagis Galiatsatos, Alexandria Soybel, Mandeep Jassal, Sergio Axel Perez Cruz, Caroline Spartin, Katie Shaw, Jodi Cunningham, Norma Fox Kanarek

**Affiliations:** 1 Division of Pulmonary and Critical Care Medicine, Department of Medicine, Johns Hopkins School of Medicine, Baltimore, MD USA; 2 Medicine for the Greater Good, Johns Hopkins Bayview Medical Center, Baltimore, MD USA; 3 Environmental Health and Engineering, Johns Hopkins School of Public Health, Baltimore, MD USA; 4 Department of Urban Residency, Johns Hopkins School of Medicine, Baltimore, MD USA; 5 The Community Builders, Inc, Cincinnati, OH USA; 6 Division of Pediatric Pulmonary, Department of Pediatrics, Johns Hopkins School of Medicine, Baltimore, MD USA; 7 Department of Oncology, Johns Hopkins School of Medicine, Baltimore, MD USA

**Keywords:** Tobacco policies, Community engagement, Population health

## Abstract

**Background:**

As a further extension of smoke-free laws in indoor public places and workplaces, the Department of Housing and Urban Development’s declaration to propose a regulation that would make housing units smoke-free was inevitable. Of note is the challenge this regulation poses to current tenants of housing units who are active smokers. We aimed to assess the efficacy of a tobacco treatment clinic in public housing. The utilization of the clinic by tenants and tenants’ respective outcomes regarding smoking status were used to determine the intervention’s effectiveness.

**Methods:**

Tobacco treatment clinics were held in two urban-based housing units for 1-year. The clinics provided on-site motivational interviewing and prescriptions for pharmacological agents if warranted. Outcomes collected include the tenants’ clinic attendance and 3- and 6-month self-reported smoking status.

**Results:**

Twenty-nine tobacco treatment clinic sessions were implemented, recruiting 47 tenants to participate in smoking cessation. The mean age of the cohort was 53 ± 12.3 years old. Of the 47 tenants who participated, 21 (44.7%) attended three or more clinic sessions. At the 3-month mark, five (10.6%) tenants were identified to have quit smoking; at 6-months, 13 (27.7%) tenants had quit smoking. All 13 of the tenants who quit smoking at the end of 6-months attended three or more sessions.

**Conclusion:**

An on-site tobacco treatment clinic to provide strategies on smoking cessation was feasible. Efforts are warranted to ensure more frequent follow-ups for tenants aiming to quit smoking. While further resources should be allocated to help tenants comply with smoke-free housing units’ regulations, we believe an on-site tobacco treatment clinic is impactful.

## Background

The Department of Housing and Urban Development’s (HUD) declaration to make HUD-supported public housing units smoke-free was inevitable [1]. Economically, prohibiting smoking in housing units could result in substantial cost savings by reducing cigarette smoke-related damage to the buildings [2, 3]. Further, the success of prior tobacco control policies indicates that such a proposal may reduce overall tobacco use, resulting in positive health outcomes and consequent medical cost savings of well over 200 million United States dollars [3–5]. However, despite these clear potential benefits - both medical and economic – the burden that a smoking ban in public housing places on active smokers should not go unrecognized.

Tobacco use and exposure, specifically smoking and secondhand smoke exposure, has experienced the smallest decline in individuals of low socioeconomic status and minorities [6–8]. Regarding associated morbidities, the number of cigarettes consumed is higher in persons with mental health illness than in the general population [9]. Such populations, both socioeconomically disadvantaged and persons with mental health morbidities, are likely to enroll in HUD-supported public housing units [10, 11]. To complicate the potential efficacy of a smoke-free housing policy, recent findings show that most active smokers do not favor smoke-free housing units [12]. Such a finding raises concerns over compliance with a smoke-free housing policy, particularly in the absence of tobacco dependence or complete cessation plans for tenants. Therefore, novel strategies are necessary to reach actively smoking tenants living in these housing units to providing educational insight into tobacco dependence management and, ultimately, complete cessation.

The objectives of this feasibility study were to assess the efficacy of a tobacco treatment clinic in public housing with regard to utilization by tenants and the ability of tenants to achieve cessation at 6-months. We hypothesized that tobacco treatment clinics in public housing units are feasible and will be well-attended by local tenants who are active smokers, resulting in quitting smoking.

## Methods

### Tobacco treatment clinics

We established clinics at two housing units in Baltimore City in 2019. The clinics were staffed on-sight with an advanced healthcare provider (physician or a nurse) and a tobacco treatment specialist. Each clinic session was 90 min long, allowing for 20 min with each tenant. Housing unit staff helped to advertise the clinics and identify active smokers of combustible tobacco. Further, the housing unit staff identified private space for interaction between healthcare professionals and the tenants. Tenants participated in the clinic voluntarily. The Institutional Review Board at Johns Hopkins School of Medicine approved the study, and all actions undertaken by the authors were in accordance with the Declaration of Helsinki.

Housing units were identified through existing partnerships. Our aim was to hold a clinical session once every two weeks. Both housing units are located in socioeconomically disadvantaged neighborhoods, as identified by their respective area deprivation index (ADI), a composite score based on census-tract level variables [13]. The ADI ranges from 1 to 100 (the higher the number, the more socioeconomically disadvantaged a neighborhood): Housing Unit 1’s ADI is 92, and Housing Unit 2’s ADI is 99. Clinical session dates were subject to cancellation by weather or holidays. Housing Unit 1 had significant construction throughout 2019, which limited tenant presence on-site.

Tenants were encouraged to come to as many clinical sessions as possible. The initial session characterized participants and their tobacco-dependence phenotypes. At the end of the first visit, tenants were introduced to current Food and Drug Administration (FDA) approved pharmacological interventions for smoking cessation and encouraged to come to additional clinical sessions. Additional clinical sessions provided ongoing tobacco-centered counseling and motivational interviewing principles established by the tobacco treatment specialist and physician. Further, at each clinical session, tenants reported whether they were current smokers or quit smoking. A tenant’s positive self-identification of actively smoking defined them as a “current smoker” [9]. Participants listed in the “quit smoking” group were defined as those who reported not smoking for more than 24-h and planned to refrain from future tobacco use [9].

If the tenant selected pharmacological agents, we encouraged obtaining the medications through two methods, with the tenant’s permission. We notified the state’s Quitline to provide nicotine replacement therapies currently available over the counter. For medications requiring a prescription, we notified the tenant’s primary care physician. Medications were dichotomized as 24-h medications, known as controllers (bupropion, nicotine replacement therapy transdermal, and varenicline), or as needed medications, known as relievers (nicotine replacement therapy gum, lozenges, inhaler, or nasal spray).

All participants were called 3-months and 6-months after their initial clinical session to discuss their smoking status and asked if they required additional resources.

### Participant characteristics

Data collected during the clinical session included age, race, and gender. Tobacco dependence-related variables included: the brand of cigarettes currently using, the age at which a tenant began to smoke, and what triggers a tenant to smoke. Triggers were identified as intrinsic (stress, boredom) and extrinsic (time of the day, social, meals). Time of the day is defined as the tenant’s reported time of use over 24 h (e.g., cigarette used during the morning or right before bed) as part of their routine. All participants were called every 4 weeks for 6 months to assess their smoking status (“quit smoking” or “current smoker”).

### Study outcomes

The primary outcome was a change in smoking status to “quit smoking” by or at the 3-month or 6-month mark. Secondary outcomes included the number of clinical sessions attended and the type of pharmacological interventions selected (e.g., only one medication or two or more medications; utilizing a controller and a reliever).

### Statistical analysis

Continuous variables were expressed as medians and interquartile ranges (IQR) or mean + standard deviation. Categorical variables were summarized as counts and percentages. For missing data, no imputation was made. Since participants in the cohort were not randomly selected, all statistics were deemed descriptive. Statistical analyses were conducted with R software (V.0.99.903).

## Results

### Demographic and clinical characteristics

Of the 47 tenants who attended one clinical session, the majority, 35 tenants (74.4%), participated from Housing Unit 2. The median age was 61.0 years old (IQR 55.0, 66.0), ranging from 22.0 to 82.0 years old. The majority of participants were female, 27 (57.4%). The median amount of cigarettes per day was 10.0 (IQR 5.0, 20.0), ranging from 5.0 to 40.0. The most used cigarette brand was Newport. A complete list of demographic and tobacco dependence variables is provided in Table 1.

**Table 1:** Summary of demographic variables. Results presented as median (IQR) where appropriate

	All Tenants (***N*** = 47)	Housing Unit 1 (***N*** = 12)	Housing Unit 2 (***N*** = 35)
**Age (years)**	61.0 (55.0, 66.0)	60.0 (58.3, 62.8)	61.0 (54.5, 66.5)
**Female**	27 (57.4%)	9 (75.0%)	18 (51.4%)
**African American**	46 (97.9%)	11 (91.7%)	35 (100%)
**Brand of Cigarettes**			
**Newport**	35 (74.5%)	9 (75.0%)	26 (74.3%)
**Kool**	5 (10.6%)	0 (0.0%)	5 (14.3%)
**Maverick**	4 (8.5%)	2 (16.7%)	2 (5.7%)
**Marlboro**	2 (4.3%)	0 (0.0%)	2 (5.7%)
**Pall Mall**	1 (2.1%)	1 (8.3%)	0 (0.0%)
**Cigarettes Per Day**	10.0 (5.0, 20.0)	15.0 (5.0, 20.0)	10.0 (5.0, 20.0)
**Age Started Smoking (years)**	15.0 (13.0, 17.5)	14.5 (13.0, 16.5)	15.0 (13.5, 17.5)
**Triggers to smoke**			
**Boredom**	20 (42.6%)	7 (58.3%)	13 (37.10%)
**Meals**	28 (59.6%)	6 (50.0%)	22 (62.9%)
**Social**	1 (2.1%)	1 (8.3%)	0 (0.0%)
**Stress**	36 (76.6%)	11 (91.7%)	25 (71.4%)
**Time of the Day**	5 (10.7%)	3 (25.0%)	2 (5.7%)

All tenants who presented to clinical sessions at the tobacco treatment clinic had attempted to quit smoking in the past. The median age of smoking initiation was 15.0 years old (IQR 13.0, 17.5), range 8.0 to 26.0 years old. Of the triggers to smoke, the most common variable identified was stress, identified by 36 tenants (76.6%). Table 1 lists a complete set of triggers that the participating tenants identified towards smoking.

### Treatment and outcomes

Twenty-nine clinical sessions were conducted over the year: 12 conducted at Housing Unit 1 and 17 at Housing Unit 2. With regard to attending sessions, 16 (34.0%) tenants participated in three or more sessions. During each session, tenants discussed their successes and struggles about quitting smoking. If they were receiving pharmacological agents to quit smoking, tolerance and side effects of the medications were reviewed. Motivational interviewing was provided at each session as well.

Of the 47 tenants, 12 (25.5%) opted not to start any pharmacological agents to assist with cessation (Table 2). The reasons for opting not to use medications included that tenants: a) felt they were already on their way to quitting and had reduced smoking behaviors at their own pace (five tenants), b) did not want to start medication (four tenants), and c) were concerned about cost (three tenants). Of the 12 tenants who opted not to receive medications, two of them participated in three or more clinical sessions (one participated in three, the other participated in five sessions total).

**Table 2:** Summary of pharmacological and counseling interventions

	All Tenants (***N*** = 47)
***Medications***	
**Daily**	
**Bupropion**	13
**NRT Transdermal**	10
**Varenicline**	7
**As Needed**	
**NRT Gum**	6
**NRT Inhaler**	1
**NRT Lozenge**	4
**NRT Nasal Spray**	10
**Amount**	
**None**	12 (25.5%)
**One**	19 (40.4%)
**Two or more**	16 (34.0%)
***Counseling***	
**Sessions Attended**	
**One**	26 (55.3%)
**Three or more**	21 (44.7%)

NRT = Nicotine Replacement Therapy

By the 6-month mark, 18 (38.3%) tenants had identified themselves as “quit smoking.” The majority of those able to quit were female (66.7%). Five of the tenants quit by the 3-month endpoint, and the rest quit by the 6-month endpoint (Table 3). Four (22.2%) patients were able to quit without any medication assistance. Of those who chose to use medications, 11 tenants (61.1%) used two or more medications. Regarding attendance of clinical sessions, 17 (94.4%) tenants attended three or more (maximum attended was seven sessions, achieved by three tenants). Of note, four tenants attended three or more clinical sessions and were unable to identify as “quit smoking” by the 6-month endpoint. These four tenants identified the following reasons they were unable to quit: increased stress (three tenants), intolerance to medications (one tenant, who used NRT transdermal and stopped using the medication 2-weeks after initiating).

**Table 3:** Characteristics of tenants who quit smoking

	All Tenants (***N*** = 18)	Quit at 3-months (***N*** = 5)	Quit at 6-months (***N*** = 13)
**Age (years)**	61.0 (51.8, 65.8)	51.0 (41.0, 60.0)	62.0 (55.0, 69.0)
**Female**	12 (66.7%)	3 (60.0%)	9 (69.2%)
**African American**	17 (94.4%)	4 (80.0%)	13 (100.0%)
**Cigarettes Per Day**	11 (6.25, 27.5)	12.0 (10.0 15.0)	10.0 (5.0, 30.0)
**Age Started Smoking (years)**	15.0 (12.5, 17.8)	14.0 (12.0, 15.0)	16.0 (14.0, 18.0)
**Amount of Medications Used**			
**None**	4 (22.2%)	1 (20.0%)	3 (23.1%)
**Only 1**	3 (16.7%)	1 (20.0%)	2 (15.4%)
**2 or more**	11 (61.1%)	3 (60.0%)	8 (61.5%)
**Medication Types**			
**Bupropion**	3	1	2
**NRT Gum**	1	1	4
**NRT Inhaler**	2	0	1
**NRT Lozenge**	5	1	1
**NRT Nasal Spray**	5	2	3
**NRT Transdermal**	5	0	5
**Varenicline**	4	2	2
**Attended 3 or more clinical sessions**	17 (94.4%)	4 (80.0%)	13 (100.0%)

NRT = Nicotine Replacement Therapy

## Discussion

In the feasibility trial of implementing an on-site tobacco treatment clinic in public housing units located in socioeconomically disadvantaged neighborhoods of an urban city, implementing the clinics was feasible. This implementation resulted in tenants actively engaged in attempting to quit smoking. Each participating housing unit was able to have one clinic session per month over the year. A significant portion of tenants who attended an initial clinic session continued to attend more clinical sessions, likely contributing to over a third of the participating tenants in the tobacco treatment clinics who were able to quit smoking by the 6-month endpoint.

In planning a smoke-free policy for public housing, planners should recognize that tenants will not merely be passive recipients of the policy and that the policy will not affect all recipients equally. A recent web-based survey identified that 73.7% of adults (out of 4203) favored smoke-free policies for housing units [12]. However, when evaluating the adults who support such a policy by smoking status, only 44.3% of current smokers favored smoke-free housing. This difference in preference may be due to concerns about achieving such a request since the housing unit is often the area a tenant smokes the most. As suggested by Berg et al., a successful complete restriction on smoking in a housing unit first requires discussions with tenants about their desire to quit [14]. This suggestion emphasizes the need to implement novel strategies, such as our on-site tobacco treatment clinic for the housing unit, the ultimate aim of which is to help tenants quit smoking.

Identifying variables associated with smoking is vital to helping patients recognize their habits and compulsions towards their tobacco dependence. Factors identified by the cohort in this trial include extrinsic (with meals, when around others) and intrinsic (boredom). One variable identified by the majority of the tenants participating in the tobacco treatment clinical sessions was stress. In Jahnel et al.’s cohort of 194 daily smokers, African American smokers reported significantly more daily stress, resulting in more daily smoking [15]. This finding is reaffirmed by our cohort, where tenants identified stress as a trigger to smoke. Tenants’ reported their experience of stress as variable and related both to personal issues (income, housing stability), as well as neighborhood-level concerns (violence and trauma of their neighborhood). Thus, moving forward, staffing tobacco treatment clinics with the appropriate behavioral health professionals may be advantageous for tenants who experience and identify stress as a catalyst to their smoking behaviors.

For those tenants who transition to the former smoker classification, the risk of relapse is considerable. Relapse rates are highest shortly after quitting and especially within the first year [16]. While many variables are associated with the likelihood of relapse to smoking, the two that warrant further emphasis for this cohort include socioeconomic status and age of smoking initiation. First, lower socioeconomic status has been associated with higher rates of relapse [17, 18]. Several reasons may explain this association, such as the increased prevalence of tobacco retail stores in neighborhoods experiencing greater poverty [19]. Our cohort’s housing units reside in socioeconomically disadvantaged neighborhoods as identified by their respective area deprivation index. Second, starting age of smoking is associated with relapse, whereby the younger the age, the higher the chance of relapse [20, 21]. In our tenant cohort, the median age of starting to smoke was 15.0 years old. Therefore, any strategies aimed at tenants of public housing to quit smoking should also focus on preventing relapse, as these variables are likely to be present in other cohorts of tenants residing in public housing. A tobacco treatment clinic could provide a sustainable advantage by ensuring patients quit and monitoring patients for potential future relapse.

Several limitations must be taken into account for this study. First, we relied on self-reporting of smoking status by the 6-month mark. Although self-reported smoking status is generally valid when compared with assessments of smoking status through biological markers, further investigation is needed to determine overall accuracy [22]. Second, the clinic was not appropriately staffed to handle cost-effectiveness issues regarding medications discussed during each session. Several tenants reported the issue of affordability as a reservation to pursuing pharmacological interventions. Future improvements with a tobacco treatment clinic should focus on assuring case managers or social workers are part of the staff to assure cost-effectiveness of the clinic recommendation. Finally, those implementing these findings should recognize that a tenant’s time residing in a particular housing unit may be brief. While our tobacco treatment clinic cohort resided within the housing units throughout the 6-months of follow-up, discussions on guaranteeing tenants who leave housing units continue managing their tobacco dependence are necessary.

## Conclusion

In this feasibility trial of tobacco treatment clinics staffed and implemented on-site in housing units, the clinics were both feasible and advantageous for tenants, with many attending three or more sessions and ultimately quitting smoking. A significant number of participating tenants utilized the clinics’ access to immediate healthcare services and ongoing counseling. If housing units attempt to implement smoke-free policies, having on-site tobacco treatment clinics staffed with health care professionals and tobacco treatment specialists may be a meaningful and impactful strategy for their respective tenants.

## Data Availability

All materials and data are available by request through the corresponding author (Panagis Galiatsatos, MD, MHS).

## Abbreviations

HUD: Department of Housing and Urban Development
ADI: Area Deprivation Index
IQR: Interquartile ranges
NRT: Nicotine replacement therapy
